# Necrotizing Crescentic Glomerulonephritis Complicating Bivalvular Bacterial Endocarditis

**DOI:** 10.7759/cureus.2520

**Published:** 2018-04-23

**Authors:** Arsalan Talib Hashmi, Muhammad Khalid, Husnain Waseem, Asiya Batool, Jignesh Patel, Stephan Kamholz

**Affiliations:** 1 Internal Medicine, Maimonides Medical Center, Brooklyn, USA; 2 Department of Internal Medicine, East Tennessee State University; 3 Internal Medicine, Maimonides Medical Center, New York, USA; 4 Internal Medicine, Jinnah Hospital Lahore (JHL)/Allama Iqbal Medical College (AIMC), Lahore, Pakistan.; 5 Department of Cardiology, Maimonides Medical Center, New York, USA; 6 Chair, Department of Medicine, Maimonides Medical Center, New York, USA

**Keywords:** infective endocarditis, crescentic glomerulonephritis, end stage renal disease

## Abstract

In the setting of an increasing incidence of endocarditis in the United States, we report a patient with necrotizing crescentic glomerulonephritis (GN) associated with native valve bacterial endocarditis due to Streptococcus parasanguinis. He was started on appropriate antibiotic treatment and subsequent blood cultures showed no growth. However, due to continuing decline in kidney function, immunosuppressive therapy was started. Despite immunosuppressive therapy and antibiotics, renal function did not improve and chronic hemodialysis was required. Due to rarity of condition, there are no definite treatment guidelines available. Antibiotics, steroids, immunosuppressive agents can be of help in most cases. Further research in this regard may help with early diagnosis and better treatment modalities.

## Introduction

Necrotizing crescentic glomerulonephritis (GN) is one of the uncommon presentations of GN complicating endocarditis. Clinical presentation is diverse and ranges from asymptomatic to gross hematuria, nephrotic or nephritic syndrome and acute decline in kidney functions. Diagnosis needs serum complement levels, immunological workup and kidney biopsy. We report a rare case of crescentic GN complicating bivalvular bacterial endocarditis who failed treatment with antibiotics, steroids, and immunosuppression.

## Case presentation

A 68-year-old Caucasian man presented with generalized weakness, dizziness without syncope, polyuria, and dyspnea on exertion. He had a past medical history of hypertension, hyperlipidemia, and coronary artery disease. Physical examination was as follows: temperature 99.3°F, pulse 84 per minute, blood pressure 168/80 mmHg, respiratory rate 18 per minute. A grade IV/VI systolic murmur was heard over the apex radiating to left axilla and back, and a grade III/VI systolic murmur was best heard at the aortic area, bibasilar crackles, hepatomegaly and pitting edema of the bilateral lower extremities were noted. Laboratory data included hemoglobin of 6.5 g/dL and blood urea nitrogen (BUN)/creatinine 71 md/dL/6.3 mg/dL, white blood cell, platelet count and lactate dehydrogenase (LDH) levels were normal. Two months previously, hemoglobin and renal function studies were normal. Urinary protein excretion was increased, but not in the nephrotic range (Microalbumin/Cr ratio = 2.00). Hepatitis B and C serology, antineutrophil cytoplasmic antibodies (ANCA), antinuclear antibody (ANA), SSA, SSB, antistreptolysin O, and anti-glomerular basement membrane (GBM) antibodies were negative and C4 complement level was normal, rheumatoid factor (RF) was 2048 IU/M and serum C3 level was 65 mg/dL (ref 80-180 mg/dL). Renal ultrasound was normal. Complete evaluation for multiple myeloma was negative.

**Figure 1:**
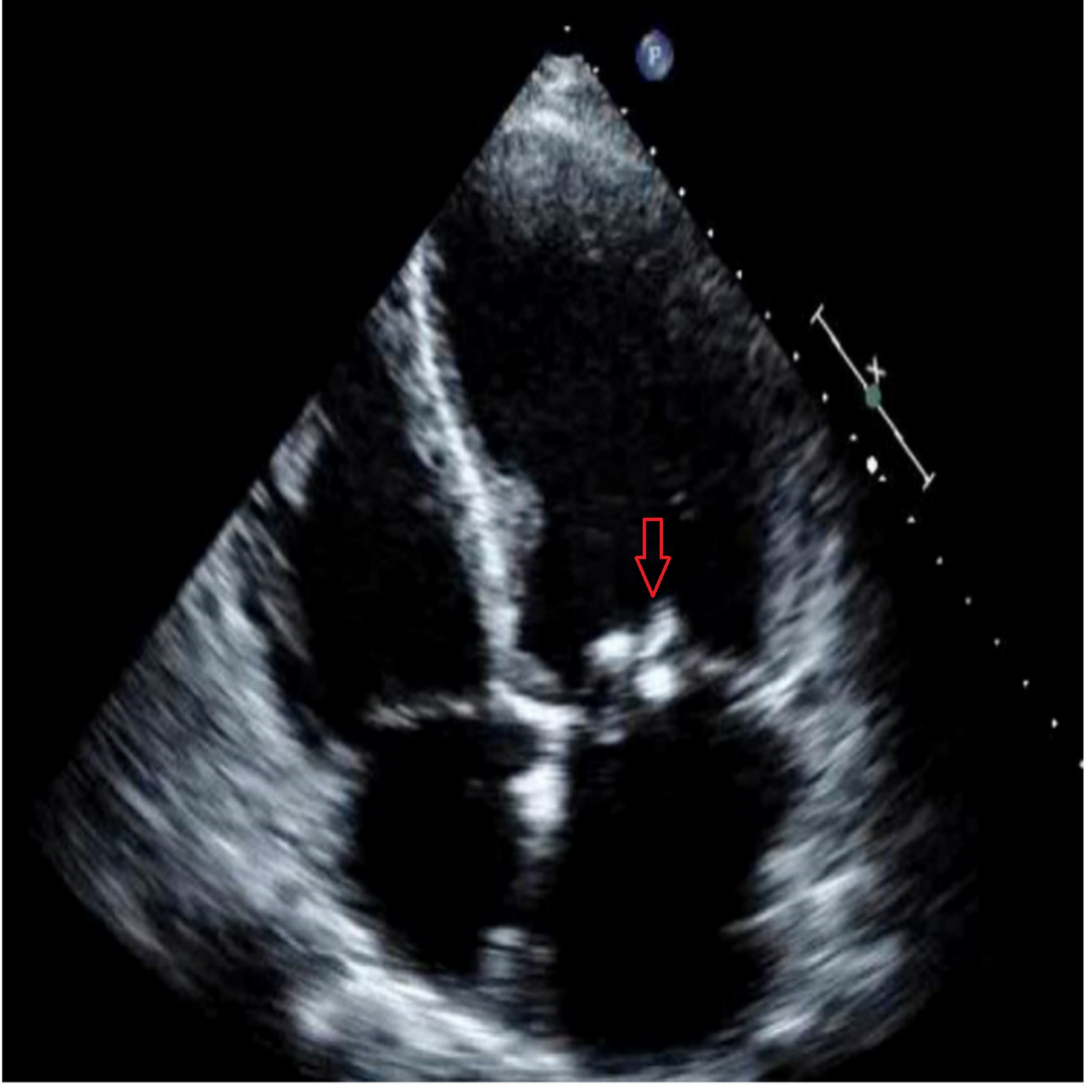
Transthoracic echocardiogram showing vegetations on mitral valve.

Transthoracic echocardiogram demonstrated severe mitral regurgitation and multiple hyperechoic masses on the tips of both mitral leaflets with a small mobile mass on the posterior mitral leaflet (Figure 1).

**Figure 2:**
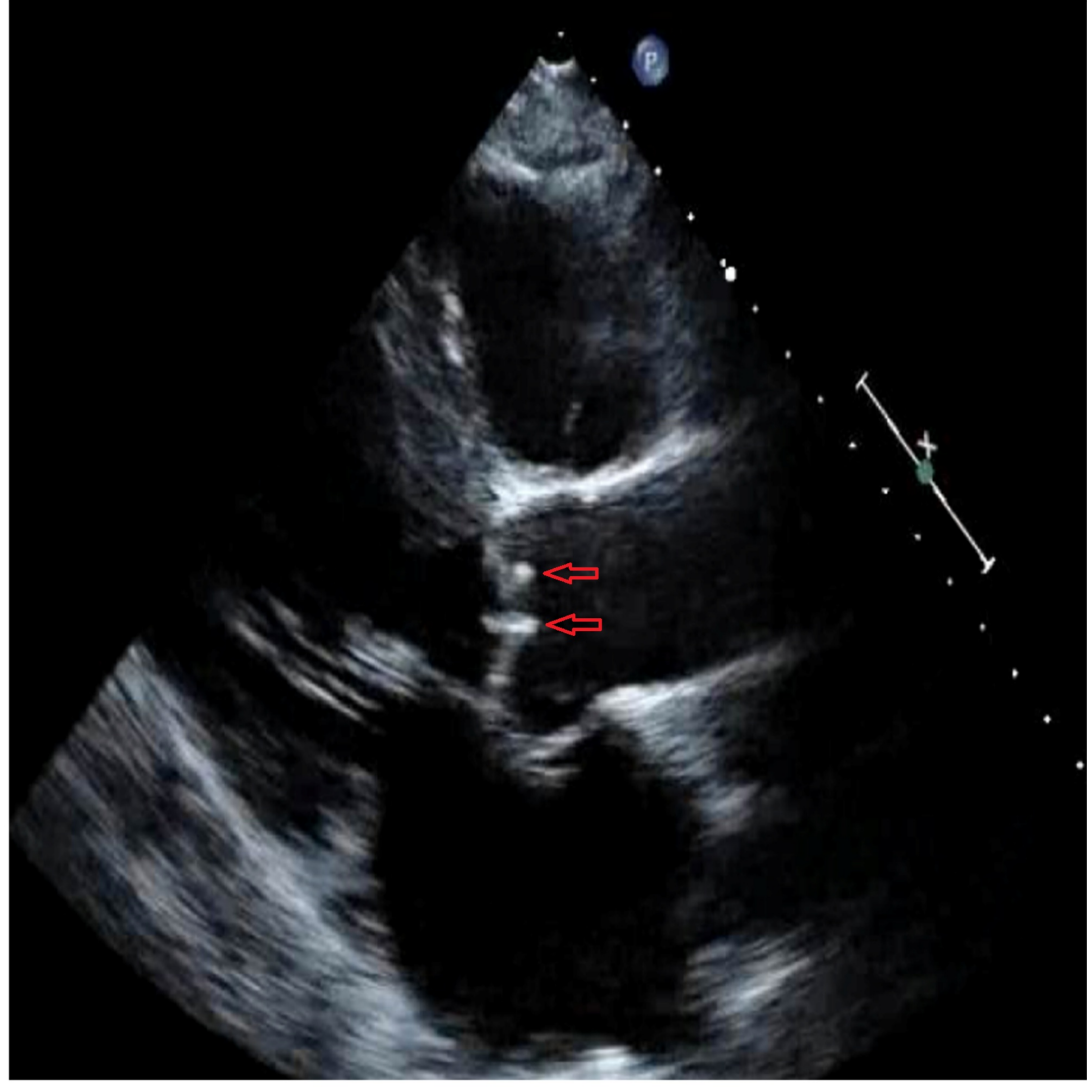
Transthoracic echocardiogram showing vegetations on aortic valve.

It also demonstrated aortic regurgitation and a mobile echogenic structure (4 mm x 4 mm), attached to ventricular side of aortic valve (Figure 2).

**Figure 3:**
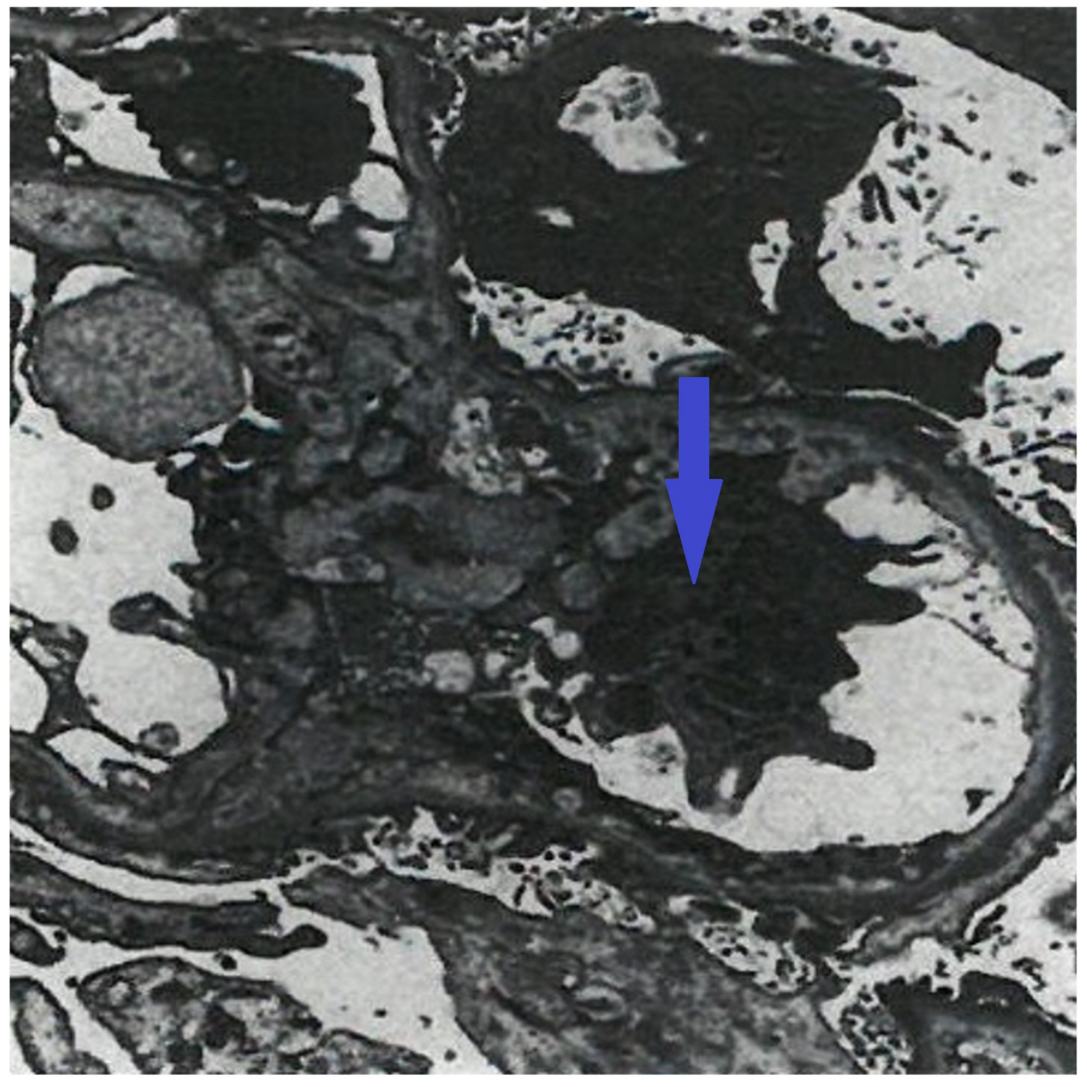
Electron microscopy showing crescent formation.

Subsequently, Streptococcus parasanguinis was isolated from blood cultures. The organism was sensitive to penicillin and ceftriaxone. Antibiotics treatment for endocarditis was initiated. Hemodialysis was required due to worsening kidney function BUN/Cr 114 mg/dL/10.3 mg/dL. Electron microscopy of renal biopsy showed crescent formation (Figure 3).

**Figure 4:**
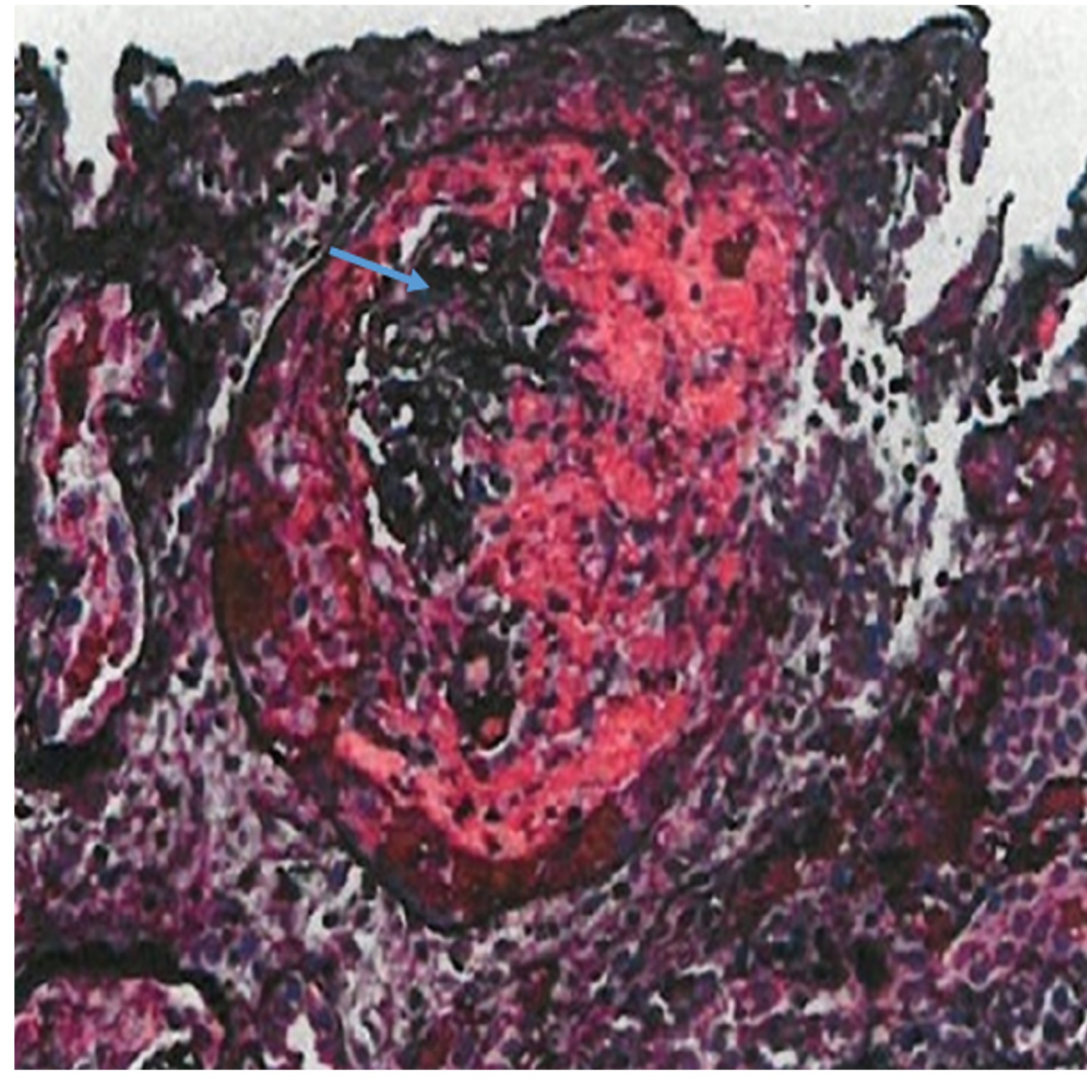
Immunofluorescence showing immune complex deposition.

Immunofluorescence showed immune complex deposition (Figure 4).

Despite antimicrobial therapy, there was no improvement in kidney function, and the patient remained dialysis dependent. Corticosteroids and cyclophosphamide were administered. Double valve replacement surgery was performed because of severe aortic and mitral valve regurgitation. Echocardiogram after surgery did not demonstrate mitral or aortic valve regurgitation, and the bioprosthetic mitral and aortic valves were normally functioning. The patient remained on dialysis despite antibiotics, steroids and cyclophosphamide. An arteriovenous (AV) fistula was placed in the left arm and maintenance hemodialysis was recommended three times per week.

On follow-up at one year, the patient remained on hemodialysis in otherwise stable condition.

## Discussion

Glomerulonephritis often complicates bacterial endocarditis. The most frequently observed renal histopathology is diffuse proliferative and exudative glomerulonephritis [1-2]. Staphylococci and streptococci are the most common bacterial organisms associated [3], and staphylococcus most commonly affects prosthetic valves while streptococci infect native valves. Rapidly progressive glomerulonephritis (RPGN) is uncommon in patients with bacterial endocarditis. It is characterized by rapid decline in kidney function and is usually a poor prognostic sign. Other prognostic factors are congestive heart failure and stroke.

Our patient has type III pauci-immune RPGN, which was caused by the endocarditis, with a compatible clinical picture and pathological confirmation by kidney biopsy which did not reveal glomerular complement or immune deposits on immunofluorescent staining and electron microscopy. Glomerular crescents were identified. Antinuclear cytoplasmic antibody determinations were negative.

Endocarditis associated with pauci-immune necrotizing crescentic glomerulonephritis is rarely reported, thus are no guidelines for definite treatment after antibiotic and corticosteroid therapy has failed. There are occasional reports of successful treatment with plasmapheresis after failed treatment with antibiotics and steroids for endocarditis associated crescentic glomerulonephritis due to Streptococcus parasanguinis [4]. Most patients usually had improvement in kidney function with antibiotics, surgical valve replacement and short course steroid therapy or immunosuppressive treatment [5].

The clinical presentation of glomerulonephritis is very diverse and ranges from asymptomatic to gross hematuria, nephrotic or nephritic syndrome and acute decline in kidney functions associated with worse outcomes.

Diagnosis needs serum complement levels, immunological workup with serology and kidney biopsy.

## Conclusions

Because of the rare presentation, there are no definite treatment guidelines available for the treatment of endocarditis-related glomerulonephritis leading to end-stage renal disease. Antibiotics are the mainstay of treatment. Short course of steroids and immunosuppressive agents should be used in patients who are unresponsive to antibiotics. Plasmapheresis and hemodialysis should be considered in patients with worsening renal function.
